# Raman spectroscopy as an alternative approach for prediction of silicate mineral content in sedimentary rocks

**DOI:** 10.1038/s41598-025-32826-w

**Published:** 2025-12-31

**Authors:** Zuzana Pěgřimočová, Michal Ritz

**Affiliations:** Department of Chemistry and Physico-Chemical Processes, Faculty of Materials Science and Technology, VSB-Technical University of Ostrava, 17. listopadu 2172/15, Ostrava-Poruba, 708 00 Czech Republic

**Keywords:** Multivariate calibration, Silicate minerals, Mineral composition, Semi-quantitative analysis, Geology, Mineralogy, Petrology, Analytical chemistry

## Abstract

This work presents Raman spectroscopy as an alternative approach for rapid prediction of silicate mineral content in sedimentary rocks. Raman spectra of clay shale and claystone samples were measured by dispersive Raman spectrometer using the excitation laser wavelength of 633 nm. Due to the presence of strong fluorescence and overlapping bands in Raman spectra of claystones and clay shales, partial least squares regression (PLSR) was employed as a multivariate calibration method for semi-quantitative estimation of silicate mineral content. The PLSR models were developed for four predominant silicate minerals of claystones and clay shales – chlorite, muscovite, quartz and albite. The correlation coefficients for calibration and validation samples were > 0.91 and > 0.84, respectively. The root mean square error of prediction (RMSEP) ranged from 1.8 to 4.9 wt %. The developed PLSR models showed good predictive capabilities for control samples and the repeatability of measurements expressed as relative standard deviation (RSD) was < 20%. This approach has the potential to be used for rapid and non-destructive on-site estimation of chlorite, muscovite, quartz and albite content in samples of claystones and clay shales using mobile Raman instruments.

**Supplementary Information:**

The online version contains supplementary material available at 10.1038/s41598-025-32826-w.

## Introduction

Knowledge of mineral composition (type and amount of minerals) of rocks is essential for determining their physical and mechanical properties and subsequent applications. Moreover, quantitative mineral composition of sedimentary rocks provides information about processes like weathering, sedimentation, transportation and diagenesis^1^. Rock analysis can be performed by several techniques. These techniques include X-ray fluorescence spectrometry (XRF)^2–4^, X-ray diffraction (XRD)^1,3,5^, electron microscopy (EM)^6–8^, inductively coupled plasma-mass spectrometry (ICP-MS)^9–11^, Fourier-transform infrared (FTIR) spectroscopy^2,3,12^, Raman spectroscopy^13–15^, Mössbauer spectroscopy (for iron-bearing minerals)^16,17^ and laser-induced breakdown spectroscopy (LIBS)^18,19^.

The most powerful and widely used technique for the quantification of mineral composition of rocks is X-ray diffraction^5,20^. Nonetheless, this technique has some limitations, including laborious and time-consuming process of powdering the sample (particle-size fractionation and milling), complex interpretation of diffractograms and its unavailability for amorphous materials^21,22^. Additionally, the quantification of e.g. clay minerals remains challenging due to the high degree of structural diversity, structural disorder, various chemical composition and preferred orientation which may lead to quantitative errors^5^. As an alternative, methods of vibrational spectroscopy (FTIR and Raman spectroscopy) can be advantageously utilized for quantification of mineral composition of rocks. These methods enable non-destructive and rapid measurements with little or no sample preparation. Moreover, a very small amount of sample (a few milligrams) is sufficient for analysis^22–24^. The use of Raman spectroscopy has many advantages over much more utilized infrared spectroscopy, including its capability to measure moist samples since water is very weak Raman scatterer and the possibility to be used on-site^23–25^. Nowadays, Raman spectroscopy offers a variety of mobile instruments. These instruments include portable Raman spectrometers weighing usually around 10 kg, smaller handheld spectrometers, or even pocket-size spectrometers weighing less than 1 kg^26^. In comparison with laboratory spectrometers, mobile Raman spectrometers provide information about studied rocks in a short amount of time, but with a lower spectral resolution^27^.

The interpretation of vibrational spectra of polymineral rocks can be challenging due to the presence of strongly overlapping bands^22,28,29^. Moreover, in case of Raman spectroscopy, fluorescence can be induced while using short-wavelength excitation lasers, resulting in strong fluorescence band (which is generally several orders of magnitude larger than Raman signal) in Raman spectrum^30,31^. This fluorescence band can often cause a loss of spectral information by overlapping the Raman signal. In these cases, the use of multivariate calibration methods seems to be the solution^32^ as they do not work with single characteristic spectral band but the whole spectrum^33^.

The objective of multivariate calibration is to establish a relationship between a desired property of a sample (e.g., concentration) and its spectrum^34^. In the field of vibrational spectroscopy, partial least squares (PLS) regression is frequently used as a multivariate calibration method^35–39^. This multivariate analysis method was originally developed at the end of 1960 s by Herman Wold within the field of econometrics and later adapted for use in chemistry by Svante Wold and Harald Martens^40^. The PLS is used to establish a relationship between data matrix X (independent variables) and vector y (dependent variables). The independent variables describe the response of the instrument (FTIR or Raman spectra), while the dependent variables describe the required property (e.g. mineral content obtained using reference method). PLS can reduce the original spectral data into a smaller number of latent variables called PLS factors, which are able to describe maximum covariance between variables X (spectral data) and Y (mineral content)^36,41–43^. A mathematical and much more detailed description of the PLS modeling can be found in the relevant publications^40,44,45^.

The development of a multivariate calibration model generally consists of several steps. First, representative calibration samples must be selected and their properties determined (e.g. mineral composition using reference method). The vibrational (infrared or Raman) spectra of these calibration samples are collected. Next, the calibration model is calculated and validated using a set of validation samples or cross-validation. Finally, the calibration model is applied to analyze unknown samples^42^.

The multivariate calibration methods (mainly PLS regression) were used for the quantification of mineral composition of rocks using both FTIR spectroscopy^12,22,29,38,43,46–48^ and Raman spectroscopy^28,49,50^. However, there is a lack of studies that focus on the quantification of mineral composition of rocks using Raman spectroscopy in more complex mixtures, where the spectral bands are strongly overlapping and are affected by fluorescence. The utilization of Raman spectroscopy for quantitative purposes may offer greater potential than FTIR spectroscopy, as it is available for on-site analysis.

The objective of the present study is to investigate the potential of Raman spectroscopy in conjunction with partial least squares (PLS) regression as a rapid method for predicting the major mineral content of sedimentary rocks. Fifty-two powdered samples of clay shales and claystones were employed as calibration and validation sets to build PLSR models for prediction of chlorite, muscovite, quartz and albite content. The predictive ability of developed PLSR models was tested via analyzing six control samples. The same control samples were analyzed to test the repeatability of measurements. In terms of accuracy, the utilization of Raman spectroscopy and PLSR cannot compete with XRD analysis as it depends on the accuracy of the XRD results. However, this approach can be used for rapid and non-destructive on-site estimation of silicate mineral content in claystones and clay shales. Moreover, this approach is suitable for the analysis of samples available in very small amounts (a few milligrams). To the best of our knowledge, this is the first time when Raman spectroscopy in conjunction with partial least squares (PLS) regression has been employed to predict the silicate mineral content using sedimentary rocks as calibration and validation samples.

## Methods

### Collection of claystone and clay shale samples

Fifty-eight pulverized samples of clay shales (forty samples) and claystones (eighteen samples) were obtained from the collection of Institute of Geonics of the Czech Academy of Sciences. The clay shale samples originated from Kyjovice layers (Lower Carboniferous period of the Moravian-Silesian area). The claystone samples were collected from the lower-to-middle Cretaceous strata in the Silesian unit of the Moravian-Silesian Beskydy Mountains (Mazak and Godula formations, Štramberk area, Hradiště and Lhota formations). The X-ray diffraction (XRD) revealed that quartz, muscovite, chlorite and albite were the most abundant minerals in all the samples. The samples of claystones from Štramberk area also revealed the prevalence of calcite. Some of the samples contained dolomite, kaolinite, ankerite, pyrite, hematite, siderite, orthoclase and rutile in minor quantities. However, most of the samples fell below the detection limit for these minor minerals. For these reasons, the research was focused on the estimation of chlorite, muscovite, quartz and albite content in the samples of claystones and clay shales. The same samples have also been used in previous studies focused on the quantification of chlorite, muscovite, quartz and albite using the DRIFT and KBr pellet techniques of FTIR spectroscopy and the partial least squares regression (PLSR)^12,46^.

 Of the total number of fifty-eight samples, fifty-two samples were used for calibration and validation (XRD results are in Supplementary Information Table S1) and six samples were used as control samples (C1-C6; XRD results are in Supplementary Information Table S2). Control samples were selected in such a way that they cover the entire concentration range of analyzed minerals. To construct the PLSR model for prediction of chlorite content, thirty calibration samples (in range from 2.8 to 25.5 wt %) and nine validation samples were employed. For construction of PLSR model for prediction of muscovite content, thirty-eight calibration samples (in range from 10.5 to 56.0 wt %) and eight validation samples were employed. For construction of PLSR model for prediction of quartz content, thirty-five calibration samples (in range from 13.5 to 64.3 wt %) and eight validation samples were employed. Finally, for construction of PLSR model for prediction of albite content, twenty-six calibration samples (in range from 2.3 to 27.0 wt %) and six validation samples were employed.

### X-ray powder diffraction (XRD) analysis

Knowledge of mineral composition of the claystone and clay shale samples was important for development of multivariate calibration models and for determination of accuracy. In the case of this study, X-ray powder diffraction analysis was utilized for these purposes. XRD analysis has been previously conducted in the context of earlier studies^12,46^. XRD measurements were carried out by diffractometer ID3003 (Rich. Seifert-FPM, Germany) using the Co lamp (heated at conditions: 40 kV, 35 mA), Fe filter, 2θ/θ goniometer geometry, step size 0.05° in 2θ range and 3 s measurement time per step. For measurement and evaluation (qualitative and quantitative), the manufacturer’s RayfleX (RayfleX scanX, RayfleX Analyze and RayfleX Autoquan) software (version 2.289) was used. The content of minerals was quantified from diffraction data by Rietveld technique^51^.

### Raman measurements

Raman spectra of powdered claystones and clay shales were collected using dispersive Raman spectrometer DXR SmartRaman (Thermo Scientific, USA) equipped with 633 nm He-Ne laser, 1200 lines/mm grating, CCD detector and 180° reflective sampling accessory. The excitation laser power was set to 7 mW. The aperture, number of exposures and exposure time were as follows: 50 μm slit, 500 times and 1s. The spot size of laser was 10 μm in diameter. The spectra were obtained in the spectral range of 1900–50 cm^− 1^. Prior to measurement, the spectrometer was calibrated using polystyrene and silicon as calibration standards. Raman spectra were recorded and processed using the OMNIC 9 software (version 9.11.706, Thermo Fisher Scientific Inc., USA; https://www.thermofisher.com/). A single Raman spectrum was recorded for each sample and therefore no spectral averaging was performed. As a treatment, the fluorescence correction was used (6th order polynomial).

### Development of PLS models

 To predict the content of silicate minerals in claystones and clay shales, PLSR models were developed using the TQ Analyst 9 software (version 9.3.107, Thermo Fisher Scientific Inc., USA; https://www.thermofisher.com/) and imported into OriginPro 8 (version 8.0725, OriginLab Corporation, USA; https://www.originlab.com/) software. The PLSR analysis was performed on the acquired Raman spectra without normalization. The initial step involved the use of calibration and validation sets (samples of claystones and clay shales with known composition) to build PLSR model and evaluate performance of calibration. The validation samples were selected in a manner that ensured coverage of the concentration range of the analyzed minerals. The mineral content of calibration and validation samples was determined by XRD analysis (used as a reference method) as described in Sect. X-ray powder diffraction (XRD) analysis. The quantitative data were then assigned to the corresponding Raman spectra of calibration and validation samples. Finally, the spectral region corresponding to Raman shift of approximately 1150–100 cm^− 1^ was chosen for analysis due to the presence of bands characteristic for the studied minerals^52–54^.

To optimize the method, the outlier spectra were assessed using two diagnostic tools (both provided by the TQ Analyst software) – Spectrum Outlier (utilizing the Chauvenet’s test for outlier detection) and Principal Component Scores (PC Scores). The PLSR models were optimized by excluding spectra of samples that were affected by high level of fluorescence emission, and whose content of the target mineral was below the detection limit of the reference method (XRD). The optimal number of factors was chosen based on the PRESS (Predicted Residual Sums of Squares) diagnostic. The PRESS diagnostic is based on the relationship between the root mean square error of cross-validation (RMSECV) and number of PLS factors. An insufficient or excessive number of PLS factors can lead to suboptimal prediction because all the variances in the data may not be represented or in the second case the model begins to include noise^38^. The selected number of factors for calibration was subsequently verified by the Loading spectra diagnostic (which displays a loading spectrum for each factor).

The developed PLSR models were characterized by the following parameters: correlation coefficient (R), root mean square error of calibration (RMSEC) and root mean square error of prediction (RMSEP). The aforementioned parameters provided information on the accuracy of the method by establishing a comparison between predicted and reference values. The smaller the RMSE value, the more accurate the PLSR model is.

The calibration error of the PLSR model, expressed as the root mean square error of calibration (RMSEC), was calculated by Eq. (1).

1 $$\:RMSEC=\:\sqrt{\frac{\sum\:_{i=1}^{n}{({c}_{i,pred}-{c}_{i,ref})}^{2}}{n}}$$

Where *c*_*i, pred*_ is the mineral content of the i^th^ calibration sample predicted from the PLS model, *c*_*i, ref*_ is the mineral content of the i^th^ calibration sample obtained by reference method (in case of this study, the Rietveld method of XRD) and n is the number of calibration samples.

The validation error of the PLSR model, expressed as the root mean square error of prediction (RMSEP), was calculated by Eq. (2).

2 $$\:RMSEP=\:\sqrt{\frac{\sum\:_{i=1}^{n}{({c}_{iv,\:pred}-{c}_{iv,\:ref})}^{2}}{m}}$$

Where *c*_*iv, pred*_ is the mineral content of the i^th^ validation sample predicted from the PLS model, *c*_*iv, ref*_ is the mineral content of the i^th^ validation sample obtained by reference method (in case of this study, the Rietveld method of XRD) and m is the number of used validation samples. The calculations of these parameters were performed by the TQ Analyst software.

## Results and discussion

### Spectral features of minerals

**Fig. 1:**
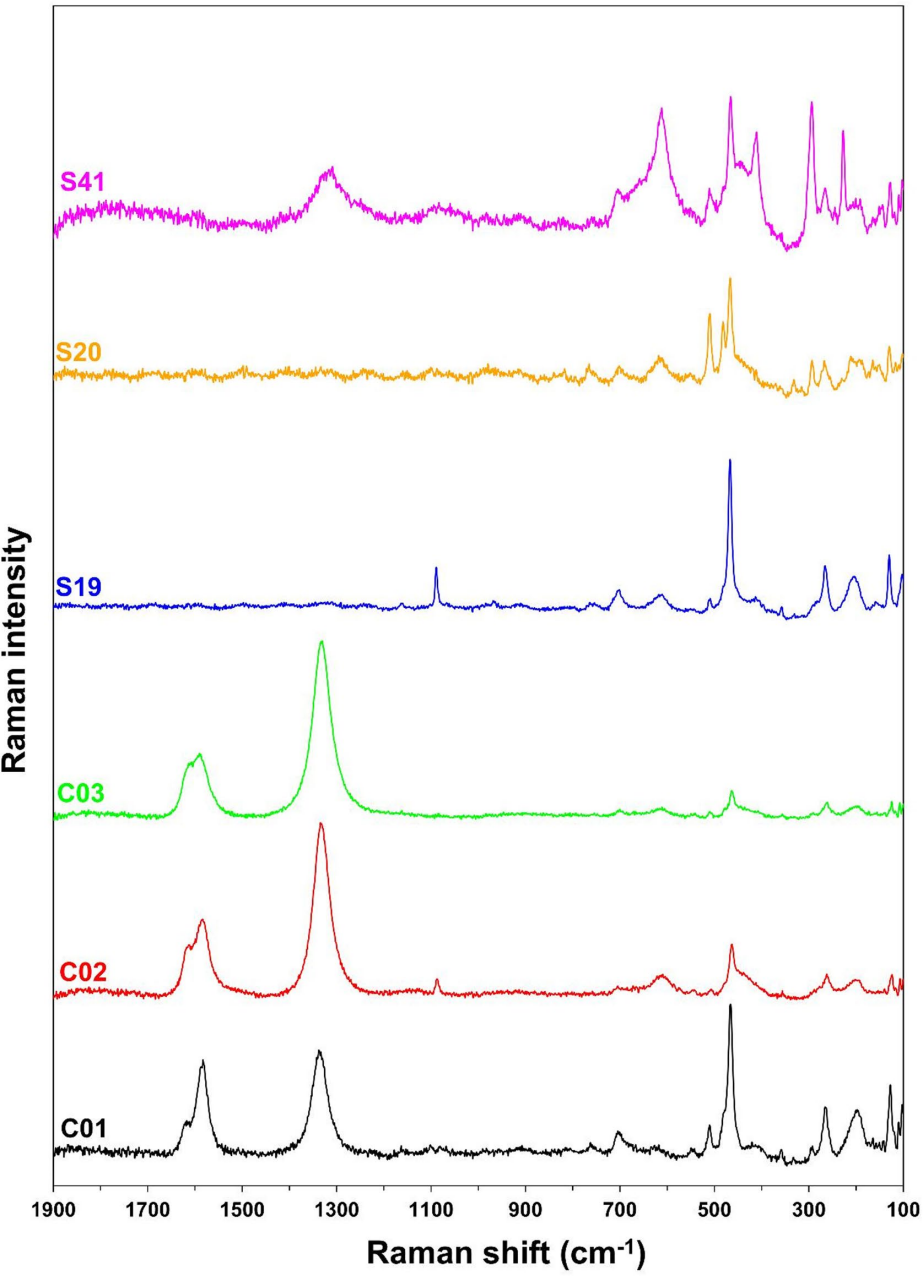
Raman spectra of selected sedimentary rock samples used in this study (after fluorescence correction).

The obtained Raman spectra contained a relatively high level of fluorescence and therefore, it would be appropriate to make measurements using a laser with wavelength of 780–1064 nm^30^. In this case, however, several expected spectral features were absent from the spectra due to the low excitation energy. For this reason, PLSR models were created from the spectra obtained using a laser with excitation wavelength of 633 nm. The Raman spectra of selected sedimentary rock samples collected using a laser with excitation wavelength of 633 nm are shown in Fig. 1.

As mentioned in Sect. Collection of claystone and clay shale samples, the studied samples were composed (based on the XRD analysis; see Table S1 and Table S2) mainly of silicate minerals such as chlorite, muscovite, quartz and albite. Other minerals (calcite, dolomite, kaolinite, ankerite, pyrite, hematite, siderite, orthoclase and rutile) were present in very small quantities and were detected only in limited number of samples. The presence of silicate minerals was also confirmed by Raman analysis, which revealed characteristic bands of chlorite (∼102 cm^− 1^, ∼359 cm^− 1^ and ∼611 cm^− 1^)^52^, muscovite (∼266 cm^− 1^, ∼411 cm^− 1^, ∼705 cm^− 1^ and ∼760 cm^− 1^)^53^, quartz (∼207 cm^− 1^ and ∼465 cm^− 1^)^50,52^ and albite (∼111 cm^− 1^, ∼166 cm^− 1^, ∼211 cm^− 1^, ∼250 cm^− 1^, ∼294 cm^− 1^, ∼482 cm^− 1^, and ∼511 cm^− 1^)^54^. Several characteristic Raman bands of these silicate minerals were found to overlap (e.g. bands characteristic for albite ∼211 cm^− 1^ and ∼250 cm^− 1^ were overlapped by ∼207 cm^− 1^ of quartz). However, the presence of several silicate mineral Raman bands in the spectra of claystones and clay shales was not always guaranteed, since they were present in some samples in small quantities and the phenomenon of fluorescence led to the loss of spectral information. Apart from Raman bands of silicate minerals, several samples exhibited Raman bands of dolomite (∼1098 cm^− 1^) and calcite (∼1088 cm^− 1^)^55^. The presence of other minerals was not detected using Raman spectroscopy. The D (∼1340 cm^− 1^), G (∼1580 cm^− 1^) and D2 (∼1620 cm^− 1^) bands, which are characteristic of carbonaceous materials^37^, were also frequently observed in the Raman spectra of studied samples.

### Performance of PLS1 models

The multivariate calibration models for content prediction of silicate minerals (chlorite, muscovite, quartz and albite) in claystones and clay shales were developed using the partial least squares (PLS) regression. The PLS can be categorized into two algorithms: PLS1 (standard algorithm) and PLS2. The difference between the two algorithms lies in the number of *y* variables that are used for PLS modelling. The PLS1 algorithm uses only one *y* variable, whereas the PLS2 algorithm is capable of modelling multiple *y* variables simultaneously. In practice this means that the PLS1 is employed for the quantification of each component individually, whereas the PLS2 for the quantification of all components simultaneously. The use of PLS2 algorithm is faster, however the number of optimum factors is calculated for all components even if the optimal number of factors may vary for each component. This fact in many cases leads to worse results^12,44,45,56^. For this reason, PLS1 algorithm was employed in the present study.

**Fig. 2:**
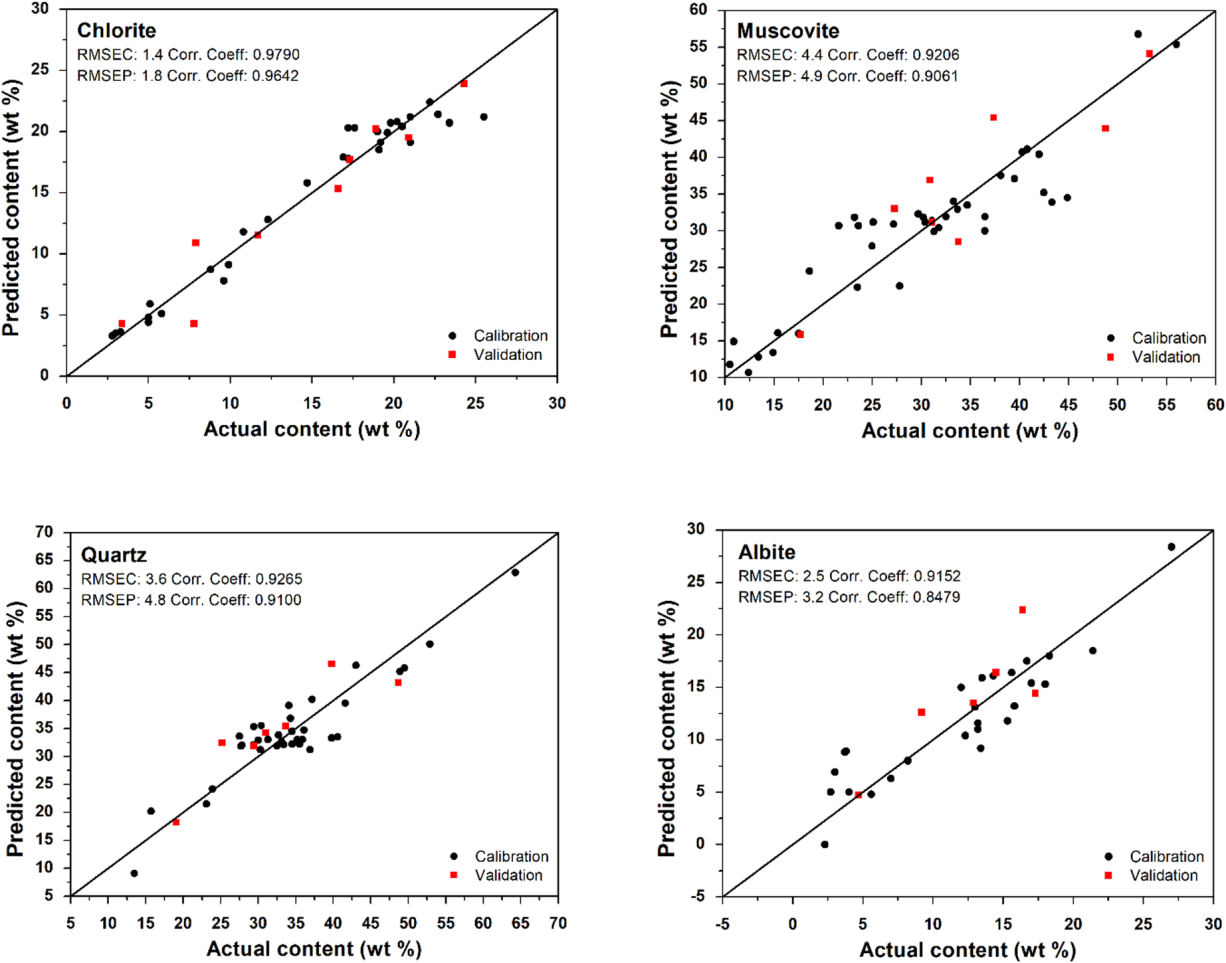
Plots of predicted (Raman spectroscopy) vs. actual (Rietveld method of XRD analysis) content for each mineral in claystones and clay shales. Calibration standards are represented by the black points and validation standards by the red points.

**Fig. 3:**
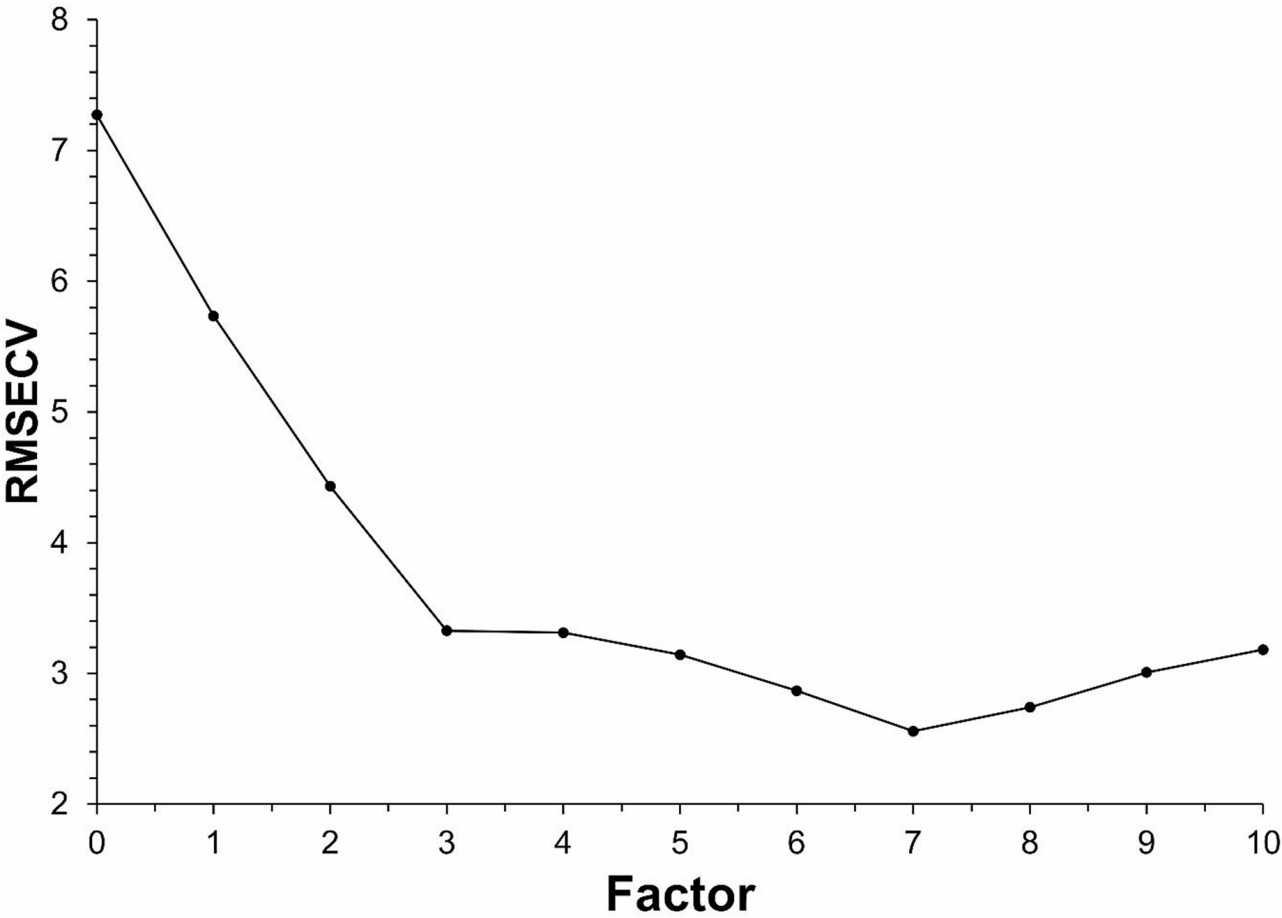
The relationship between the root mean square error of cross-validation (RMSECV) and number of PLS factors for calibration model of chlorite.

The developed PLSR models for each mineral are shown in Fig. 2. Statistical parameters characterizing the performance of the developed PLSR models, are listed in Table 1. PLSR models developed for predicting silicate mineral composition of sedimentary rocks showed good performances. The range of correlation coefficient for calibration set (R) was from 0.9152 to 0.9790, the range of correlation coefficient for validation set (R_P_) was from 0.8479 to 0.9642. RMSEC values varied between 1.4 and 4.4 wt % and RMSEP values between 1.8 and 4.9 wt %. In most cases, the RMSE values in this study were lower than those obtained from PLSR models based on FTIR spectra of claystones and clay shales from previous study. The RMSE values for albite were only slightly greater^46^. Optimal number of factors varied between 5 and 9 based on the minimum value of RMSECV, ranging from 2.6 to 7.0 wt %. As an example, the relationship between the RMSECV and number of PLS factors for calibration model of chlorite is demonstrated on Fig. 3. In this case, the minimum value of RMSECV was achieved at 7 PLS factors.

**Table 1:** Parameters of developed PLSR models for semi-quantitative estimation of silicate mineral content based on Raman spectra.

Mineral	*R*	*R*_*P*_	RMSEC (wt %)	RMSEP (wt %)	RMSECV (wt %)	Number of factors
Chlorite	0.9790	0.9642	1.4	1.8	2.6	7
Muscovite	0.9206	0.9061	4.4	4.9	7.0	9
Quartz	0.9265	0.9100	3.6	4.8	6.4	6
Albite	0.9152	0.8479	2.5	3.2	4.1	5

### Analysis of control samples (accuracy)

The accuracy of the four PLSR models to predict mineral content in unknown samples was tested using six control samples (C1-C6). The Raman spectra of those control samples were inserted into TQ Analyst software which then calculated their mineral content based on the developed PLSR models. The results of the studied silicate mineral content in the control samples predicted by PLSR models and the studied silicate mineral content in the same control samples obtained by XRD are given in Table 2. Uncertainties of mineral content predicted by PLSR models correspond to RMSEP values^22^.

**Table 2:** XRD determined vs. predicted mineral content for claystones and clay shales.

Sample	Chlorite		Muscovite		Quartz		Albite	
	XRD	PLS	XRD	PLS	XRD	PLS	XRD	PLS
C01	17.6 ± 1.8	16.1 ± 1.8	32.4 ± 1.7	33.8 ± 4.9	30.1 ± 1.2	34.7 ± 4.8	19.8 ± 1.1	18.9 ± 3.2
C02	18.0 ± 2.5	17.5 ± 1.8	25.4 ± 2.4	29.8 ± 4.9	36.9 ± 1.8	34.5 ± 4.8	14.7 ± 1.4	14.6 ± 3.2
C03	22.8 ± 2.6	19.8 ± 1.8	34.0 ± 2.8	31.5 ± 4.9	32.4 ± 1.8	33.1 ± 4.8	10.9 ± 1.6	11.6 ± 3.2
C04	5.5 ± 2.8	1.3 ± 1.8	14.4 ± 4.2	8.0 ± 4.9	27.3 ± 2.0	19.0 ± 4.8	< 1.0	N/A
C05	22.5 ± 4.8	21.2 ± 1.8	45.9 ± 4.8	32.2 ± 4.9	25.9 ± 2.7	32.3 ± 4.8	3.0 ± 2.0	8.9 ± 3.2
C06	21.6 ± 2.4	19.6 ± 1.8	31.9 ± 2.4	31.5 ± 4.9	34.0 ± 1.6	32.7 ± 4.8	12.6 ± 1.4	12.6 ± 3.2

To determine the difference between the quantity obtained using the reference method and the quantity obtained from chemometric calibration models, the following were calculated:

1. Relative error (RE)
  3 $$\:RE=\frac{\left|{c}_{i,pred}-{c}_{i,ref}\right|}{{c}_{i,ref}}\cdot\:100$$

2) Standard error of prediction (SEP)
  4 $$\:SEP=\:\sqrt{\frac{\sum\:_{i=1}^{n}({c}_{i,pred}-{c}_{i,ref}{)}^{2}}{n}}$$

In these equations, *c*_*i, pred*_ is the mineral content value of the i^th^ control sample predicted from the PLSR model, *c*_*i, ref*_ is the mineral content value of the i^th^ control sample obtained by the reference method (the Rietveld method of XRD analysis) and *n* is the number of control samples. It should be noted that the accuracy of the results obtained from developed PLSR models is inherently limited and depends on the accuracy of the reference method (in case of this study, the Rietveld method of XRD analysis). Therefore, the accuracy of the results obtained from PLSR models cannot be higher than the accuracy of the reference method.

The accuracy expressed by the relative error (RE) for each control sample is shown in Table 3. Some developed PLSR models demonstrated a lack of efficacy in describing lower content values due to a low number of calibration samples describing lower content values. A comparable phenomenon was also observed with high content values of muscovite. Consequently, samples with very low mineral content often exhibited extremely high RE values resulting in distortion of values of mean RE. For example, in case of albite, the difference (absolute error) between the value obtained by XRD (3.0 wt %) and value predicted by PLSR model (8.9 wt %) for sample C05 was 5.9 wt % and RE was 196.7%. In case of muscovite, the content value obtained by XRD was 45.9 wt % and the content value obtained from PLSR model was 32.2 wt %, this difference was even greater (13.7 wt %) but RE value was only 29.9%. The fact that RE for lower values increases rapidly is evident from the mathematical formulation. For chlorite, muscovite and quartz the mean RE was below 20%. For albite, the mean RE value was ~ 40%.

**Table 3:** Relative error (RE) values calculated for each mineral and control sample.

Samples	RE (%)			
	Chlorite	Muscovite	Quartz	Albite
C01	8.5	4.3	15.3	4.6
C02	2.8	17.3	6.5	0.7
C03	13.2	7.4	2.2	6.4
C04	76.4	44.4	30.4	N/A
C05	5.8	29.9	24.7	196.7
C06	9.3	1.3	3.8	0.0
**Mean**	19.3	17.4	13.8	41.7

The accuracy expressed by standard error of prediction (SEP) ranged from 2.4 wt % to 6.5 wt %. The best SEP value had PLSR model for the prediction of chlorite content with 2.4 wt %. In contrast, the highest value of SEP (6.5 wt %) was observed by the PLSR model of muscovite due to a large difference between the muscovite content predicted by PLSR model and muscovite content obtained using XRD in sample C05.

A comparison of the obtained results of accuracy with the previous study^46^ which was focused on the quantification of the same minerals in the same type of samples using combination of FTIR spectroscopy (DRIFT and KBr pellet techniques) and PLSR, is presented in Table 4. The standard error of prediction (SEP) values obtained in this study for chlorite, muscovite and quartz were found to be either almost identical or lower (in case of muscovite) compared to those reported in previous study^46^. In the case of albite, the SEP value was found to be higher than the SEP value reported in FTIR study^46^. The mean relative errors (RE) of chlorite, muscovite, quartz and albite were either similar or greater to those obtained by FTIR spectroscopy. This is mainly because the PLSR models developed using infrared spectra (both DRIFT and pellet technique) were more accurate (mostly in case of albite) in describing lower mineral content values. However, except for low mineral content, the accuracy of the results obtained using Raman spectroscopy was comparable to those obtained using infrared spectroscopy^46^.

**Table 4:** Comparison of accuracy of PLSR models based on Raman (current study) and FTIR spectra.

Technique	Chlorite		Muscovite		Quartz		Albite	
	SEP (wt %)	RE (%)	SEP (wt %)	RE (%)	SEP (wt %)	RE (%)	SEP (wt %)	RE (%)
Raman spectroscopy	2.4	19.3	6.5	17.4	4.8	13.8	2.7	41.7
FTIR spectroscopy (DRIFT)^46^	2.4	20.7	10.3	12.9	4.9	10.5	1.7	19.4
FTIR spectroscopy (KBr pellet)^46^	2.1	11.5	19.1	13.9	4.2	12.6	1.6	9.1

### Analysis of control samples (repeatability)

The precision of the developed PLSR models was demonstrated on the same samples that were used for accuracy testing (C01-C06). Each sample was measured ten times (10 portions of the re-homogenized same sample) under the same condition of measurement and on the same day. Measured spectra were then used for prediction of chlorite, muscovite, quartz and albite content using developed PLSR models. A relative standard deviation (RSD) was used for estimation of repeatability of measurement. The results of testing repeatability are presented in Table 5. For most of the control samples and PLSR models, the repeatability, expressed as RSD, was less than 10%, indicating that the samples were relatively homogeneous. The highest RSD values were exhibited by sample C04, particularly in the prediction of chlorite content due to its low content leading to the higher RSD values. The Raman spectrum of control sample C04 was of considerably lower quality as it was strongly affected by high level of fluorescent emission. The high RSD values may therefore indicate inappropriateness of Raman spectra of highly fluorescent samples for prediction of mineral composition using PLSR.

**Table 5:** Repeatability of mineral content prediction in claystones and clay shales using PLSR.

		Predicted mineral content (wt %)										
Mineral	Sample	1	2	3	4	5	6	7	8	9	10	RSD (%)
Chlorite	C01	12.8	15.9	16.5	17.2	15.0	14.3	16.7	13.4	15.0	15.3	9.4
	C02	15.8	13.2	17.7	15.7	17.8	15.4	16.2	17.0	14.7	16.4	8.7
	C03	19.7	19.9	16.5	19.8	18.9	18.5	19.0	18.5	16.5	17.9	6.7
	C04	−0.2	−23.7	0.7	−5.0	−4.4	−2.0	−13.0	−4.0	−7.0	−7.0	−109.4
	C05	21.2	21.2	21.2	21.3	21.1	21.2	21.2	21.4	21.3	21.2	0.4
	C06	20.3	20.1	20.1	20.1	20.4	20.0	20.4	19.7	20.4	20.0	1.3
Muscovite	C01	24.1	28.6	29.0	30.6	27.9	27.2	31.5	26.9	31.4	30.3	8.1
	C02	29.7	23.9	30.3	29.7	31.7	29.1	28.8	29.7	27.8	28.6	7.1
	C03	31.7	33.1	28.3	32.0	31.3	30.4	31.4	30.0	26.8	29.4	6.2
	C04	37.2	11.9	41.9	31.8	29.0	31.8	32.4	34.8	35.3	35.3	24.7
	C05	32.7	32.6	32.7	32.8	32.7	33.0	33.1	33.1	33.2	33.1	0.7
	C06	32.4	32.3	32.4	32.2	32.6	32.2	32.4	32.0	32.7	32.3	0.6
Quartz	C01	37.9	35.0	35.2	34.6	35.4	37.9	34.8	37.7	36.9	35.2	3.8
	C02	35.6	38.7	35.3	36.1	34.4	36.7	35.0	34.7	36.9	35.0	3.6
	C03	34.1	34.0	35.6	33.4	34.8	34.7	34.0	35.2	36.8	34.9	2.8
	C04	28.2	2.5	30.5	21.2	22.5	18.5	22.5	21.5	23.5	22.0	35.1
	C05	31.6	31.6	31.7	31.5	31.6	31.6	31.6	31.4	31.6	31.6	0.2
	C06	32.6	32.7	32.6	32.5	32.3	32.6	32.6	32.9	32.3	32.4	0.6
Albite	C01	31.9	24.2	23.0	20.3	27.5	28.5	21.9	30.5	22.6	24.5	15.3
	C02	15.0	21.7	13.8	11.9	12.5	15.8	18.3	15.2	15.0	16.9	18.3
	C03	11.7	11.7	14.5	12.8	10.2	12.7	11.7	9.7	14.3	14.5	13.8
	C04	N/A	N/A	N/A	N/A	N/A	N/A	N/A	N/A	N/A	N/A	N/A
	C05	10.2	10.4	10.5	9.9	10.4	9.8	9.7	9.8	9.4	9.9	3.6
	C06	10.2	10.4	10.5	11.1	10.6	10.9	11.2	11.9	10.6	10.8	4.5

## Conclusions

In this paper, Raman spectroscopy was employed in combination with partial least squares regression (PLSR) for semi-quantitative estimation of silicate mineral (chlorite, muscovite, quartz and albite) content in sedimentary rocks. Samples of claystones and clay shales with known composition (determined by XRD) were utilized as calibration and validation samples in the process of PLSR models development. The correlation coefficients for calibration and validation samples were > 0.91 and > 0.84, respectively. RMSEC values varied between 1.4 and 4.4 wt % and RMSEP values between 1.8 and 4.9 wt %. The developed PLSR models showed relatively good predictive capabilities for control samples. However, some developed PLSR models demonstrated a lack of efficacy in describing lower and higher content values due to a low number of calibration samples representing lower or higher content values. The accuracy was expressed as a standard error of prediction (SEP) which ranged from 2.4 wt % to 6.5 wt % and as a mean relative error (RE) which was < 20% for chlorite, muscovite and quartz and ~ 40% for albite. The repeatability of measurements expressed as relative standard deviation (RSD) was < 20%. The results obtained using Raman spectroscopy were comparable to those obtained using FTIR spectroscopy^46^. In terms of accuracy, the utilization of Raman spectroscopy and PLSR presented in this study cannot compete with XRD analysis (as it depends on the accuracy of the XRD results). However, this approach has the potential to be used for rapid and non-destructive on-site estimation of chlorite, muscovite, quartz and albite content in samples of clay shales and claystones. Moreover, this approach is also suitable for the analysis of samples that are available in very small amounts.

## Supplementary Information

Below is the link to the electronic supplementary material.

Supplementary Material 1

## Data Availability

The datasets generated during and analysed during the current study are available at 10.5281/zenodo.17916073.
