# Supplementary material for: Raman spectroscopy as an alternative approach for prediction of silicate mineral content in sedimentary rocks
Source: Sci Rep. 2025 Dec 31;16:2891. doi: 10.1038/s41598-025-32826-w (PMC12830797; doi:10.1038/s41598-025-32826-w)
Supplement: Supplementary file 1 — Supplementary Material 1 [file 41598_2025_32826_MOESM1_ESM.pdf]

Supplementary Information

Raman spectroscopy as an alternative approach for prediction of silicate mineral content in sedimentary rocks

Zuzana Pěgřimočová[1], Michal Ritz[1,*]

[1]*Department of Chemistry and Physico-Chemical Processes, Faculty of Materials Science and Technology, VSB-Technical University of Ostrava, 17. listopadu 2172/15, 708 00 Ostrava-Poruba, Czech Republic*

[*]michal.ritz@vsb.cz

Table S1 List of samples used as calibration and validation sets. Same samples have also been used in previous studies [1,2].

Sample	Type of sedimentary rock	Chlorite		Muscovite		Quartz		Albite	
		content (wt %)	±	content (wt %)	±	content (wt %)	±	content (wt %)	±
**S01**	claystone	8.2	*1.9*	40.3	*5.1*	44.9	*4.8*	3.0	*0.8*
**S02**	claystone	9.7	*6.3*	23.5	*2.4*	64.3	*4.8*	2.5	*0.6*
**S03**	claystone	3.0	*1.5*	37.4	*2.9*	52.9	*2.5*	6.7	*1.1*
**S04**	claystone	7.9	*2*	48.8	*2.5*	34.1	*1.6*	9.2	*1.2*
**S05**	claystone	5.0	*1.4*	40.8	*3.9*	48.9	*1.8*	8.2	*1.7*
**S06**	claystone	7.8	*1.8*	53.3	*5.7*	29.4	*1.6*	4.7	*0.9*
**S07**	claystone	8.8	*2.6*	56.0	*5.1*	36.1	*1.5*	5.6	*1*
**S08**	claystone	5.1	*1.4*	52.1	*5.4*	33.2	*1.7*	4.0	*0.8*
**S09**	claystone	22.2	*3*	39.5	*3*	19.5	*1.3*	18.9	*1.6*
**S10**	clay shale	15.6	*1.7*	27.8	*1.8*	39.8	*1.4*	16.8	*1.1*
**S11**	clay shale	9.1	*2.1*	21.6	*1.8*	53.8	*1.9*	15.6	*1.2*
**S12**	clay shale	10.4	*2*	23.2	*1.9*	48.4	*1.6*	18.0	*1.1*
**S13**	clay shale	17.2	*1.8*	31.1	*2.6*	26.3	*1.4*	17.9	*1.4*
**S14**	clay shale	17.2	*2.1*	53.8	*2.2*	25.2	*1.3*	3.7	*1.2*
**S15**	clay shale	22.7	*2.4*	25.1	*2.4*	30.3	*1.4*	21.4	*1.5*
**S16**	clay shale	11.6	*2*	47.6	*2*	36.9	*1.5*	3.8	*1.1*
**S17**	clay shale	29.6	*2.8*	43.3	*2.9*	20.2	*1.5*	7.0	*1.5*
**S18**	clay shale	19.2	*2.6*	33.7	*2.8*	28.1	*1.5*	19.0	*1.5*
**S19**	clay shale	3.3	*1.3*	38.1	*2*	43.0	*1.7*	11.2	*1.3*
**S20**	clay shale	9.6	*2.2*	18.6	*2.3*	30.4	*1.4*	41.5	*1.9*
**S21**	clay shale	12.3	*3.3*	25.0	*2.6*	49.5	*2.1*	2.3	*1.1*
**S22**	clay shale	20.2	*2.3*	33.3	*2.4*	32.5	*1.6*	13.4	*1.3*
**S23**	clay shale	25.5	*2.3*	42.5	*2.5*	27.5	*1.4*	2.7	*1.2*
**S24**	clay shale	19.8	*2.3*	50.4	*2.3*	29.4	*1.5*	0.4	*0.8*
**S25**	clay shale	19.6	*2.7*	30.4	*2.7*	33.1	*1.9*	13.2	*1.6*
**S26**	clay shale	20.9	*2.4*	31.1	*2.5*	35.2	*1.7*	12.9	*1.4*
**S27**	clay shale	19.1	*3.6*	36.5	*3.6*	30.0	*2.3*	14.3	*2.1*
**S28**	clay shale	23.4	*3*	36.5	*3*	27.8	*1.8*	12.3	*1.7*
**S29**	claystone	<1.0	-	2.2	*2.4*	48.7	*1.8*	<1.0	-
**S30**	claystone	3.4	*2.6*	17.7	*5.7*	19.1	*1.7*	1.5	*1.4*
**S31**	claystone	5.0	*1.9*	12.4	*2.4*	15.7	*1.2*	<1.0	-
**S32**	claystone	2.8	*2.5*	17.5	*4.5*	37.2	*2.4*	<1.0	-
**S33**	claystone	3.8	*2.1*	10.5	*3*	13.5	*1.1*	<1.0	-
**S34**	claystone	5.8	*2.1*	14.9	*2.1*	41.6	*2*	2.3	*1.9*
**S35**	claystone	<1.0	-	10.9	*2.1*	23.1	*1.4*	<1.0	-

**S36**	claystone	<1.0	-	13.4	*2*	34.5	*1.7*	<1.0	-
**S37**	claystone	5.4	*2.1*	15.4	*4.2*	23.9	*1.7*	<1.0	-
**S38**	clay shale	13.9	*4.2*	33.8	*3.6*	35.9	*2.3*	16.4	*1.7*
**S39**	clay shale	10.8	*3*	9.9	*2.7*	52.3	*2.5*	27.0	*2.3*
**S40**	clay shale	24.3	*3.9*	30.9	*3.3*	29.4	*2*	14.5	*1.7*
**S41**	clay shale	9.9	*2.3*	42.0	*2.6*	34.3	*1.6*	13.5	*1.4*
**S42**	clay shale	17.3	*2.1*	27.3	*2.6*	32.7	*1.5*	17.3	*1.4*
**S43**	clay shale	17.6	*2.1*	34.7	*2.2*	34.5	*1.4*	13.2	*1.3*
**S44**	clay shale	21.0	*4.8*	44.9	*4.8*	27.7	*2.8*	3.0	*2.1*
**S45**	clay shale	11.7	*5.1*	30.0	*5.1*	5.3	*1.2*	1.0	*1.4*
**S46**	clay shale	18.9	*2.3*	32.5	*2.4*	33.4	*1.6*	15.3	*1.5*
**S47**	clay shale	16.9	*2.4*	23.6	*2.4*	40.6	*1.9*	13.0	*1.4*
**S48**	clay shale	21.0	*2.4*	27.2	*2.3*	39.8	*1.7*	12.0	*1.3*
**S49**	clay shale	14.7	*2.2*	31.8	*2.8*	31.0	*1.5*	18.3	*1.5*
**S50**	clay shale	16.6	*2.2*	31.3	*2.6*	33.7	*1.5*	16.7	*1.5*
**S51**	clay shale	20.5	*2.2*	30.2	*2.5*	31.3	*1.4*	17.0	*1.4*
**S52**	clay shale	19.0	*2.6*	29.7	*2.6*	35.5	*1.7*	15.8	*1.5*

Samples S04, S06, S26, S30, S40, S42, S45, S46, S50 were used for validation of PLSR model for prediction of chlorite content; S03, S04, S06, S26, S30, S38, S40, S42 were used for validation of PLSR model for prediction of muscovite content; S01, S10, S14, S29, S30, S40, S49, S50 were used for validation of PLSR model for prediction of quartz content S04, S06, S26, S38, S40, S42 were used for validation of PLSR model for prediction of albite content.

Table S2 List of control samples. Same samples have also been used in previous studies [1,2].

Sample	Type of sedimentary rock	Chlorite		Muscovite		Quartz		Albite	
		content (wt %)	±	content (wt %)	±	content (wt %)	±	content (wt %)	±
**C01**	clay shale	17.6	*1.8*	32.4	*1.7*	30.1	*1.2*	19.8	*1.1*
**C02**	clay shale	18.0	*2.5*	25.4	*2.4*	36.9	*1.8*	14.7	*1.4*
**C03**	clay shale	22.8	*2.6*	34.0	*2.8*	32.4	*1.8*	10.9	*1.6*
**C04**	claystone	5.5	*2.8*	14.4	*4.2*	27.3	*2.0*	<1.0	-
**C05**	clay shale	22.5	*4.8*	45.9	*4.8*	25.9	*2.7*	3.0	*2.0*
**C06**	clay shale	21.6	*2.4*	31.9	*2.4*	34.0	*1.6*	12.6	*1.4*

References

[1] Ritz, M., Vaculíková, L., Plevová, E., Matýsek, D. & Mališ, J. Determination of the predominant minerals in sedimentary rocks by chemometric analysis of infrared spectra. *Clays Clay Miner.* **60**, 655 – 665. https://doi.org/10.1346/CCMN.2012.0600609 (2012).

[2] Ritz, M., Vaculíková, L., Plevová, E., Matýsek, D. & Mališ, J. Determination of chlorite, muscovite, albite and quartz in claystones and clay shales by infrared spectroscopy and partial least-squares regression. *Acta Geodyn. Geomater.* **9**, 511 – 520. https://www.irsm.cas.cz/materialy/acta_content/2012_04/9.Ritz.pdf (2012).
